# Mutagenesis selection and large-scale cultivation of non-green *Chlamydomonas reinhardtii* for food applications

**DOI:** 10.3389/fnut.2024.1456230

**Published:** 2024-09-25

**Authors:** Gang Cao, Kun Hu, Zhewen Hu, Qianlong Wu, Siyuan Liu, Xiaoping Chen, Xiangrui Meng, Zhangfeng Hu, Li Feng

**Affiliations:** ^1^Institute of Microalgae Synthetic Biology and Green Manufacturing, School of Life Sciences, Jianghan University, Wuhan, China; ^2^Engineering Training Center, Jianghan University, Wuhan, China; ^3^School of Medicine, Jianghan University, Wuhan, China; ^4^Hubei Engineering Research Center for Protection and Utilization of Special Biological Resources in the Hanjiang River Basin, School of Life Sciences, Jianghan University, Wuhan, China; ^5^Hubei Key Laboratory of Environmental and Health Effects of Persistent Toxic Substances, Jianghan University, Wuhan, China

**Keywords:** *Chlamydomonas reinhardtii*, ARTP mutagenesis, chlorophyll deficiency, high-density heterotrophic cultures, nutritional applications

## Abstract

**Background:**

The green alga *Chlamydomonas reinhardtii* is an accepted food ingredient in the United States of America (United States), the European Union, Singapore, and China. It can be consumed in unlimited quantities. As this alga is rich in nutrients, proteins, and rough polysaccharides and contains a balanced proportion of various amino acids, it is an excellent raw material for food production. Although various edible brown and green algae are available on the market, their color and strong grassy flavor have constrained their popularity among consumers, thereby limiting their application in food additives and animal feed.

**Methods:**

Chlorophyll-deficient *C. reinhardtii* mutants were developed using atmospheric and room temperature plasma (ARTP) technology.

**Results:**

A yellow-colored *C. reinhardtii* variant (*A7S80*) cultivated in dark conditions was isolated. This light-sensitive variant has a mutation in the *chlM* gene, and it can grow heterotrophically using acetate as a carbon source.

**Conclusion:**

Compared to wild-type *C. reinhardtii*, *A7S80* has significantly lower chlorophyll levels, reduced grassy flavor, and more diverse pigments, with considerable potential for commercial application in human and animal food production, as well as in pharmaceutical and cosmetic industries.

## 1 Introduction

Microalgae are autotrophic microorganisms that grow in fresh and marine waters and soil ecosystems. They are coveted for use in nutraceuticals and as a dietary supplement. In addition to proteins, microalgae are rich in biologically active components such as *β*-carotene, eicosapentaenoic acid (EPA), and docosahexaenoic acid (DHA) (1). As a common model organism, *Chlamydomonas reinhardtii* is often used for research on topics, such as photosynthetic systems, flagellar assembly, and recombinant protein factories (2–4). Recently, *C. reinhardtii* has been acknowledged by the United States Food and Drug Administration (FDA) as “Generally Regarded as Safe” (GRAS) for human consumption, highlighting its significant potential as a functional food. This alga has also been officially approved by the National Health Commission of China. It is recognized as a safe food ingredient in the European Union and Singapore, and it is also permitted for sale in Hong Kong.

*Chlamydomonas reinhardtii* is widely distributed in nature, grows rapidly, and is highly adaptable. It can be photosynthetically autotrophic or heterotrophic, using organic carbon sources, and the cultivation process is straightforward, earning it the moniker “photosynthetic yeast” (5). This alga, rich in nutrients, boasts a dry-weight protein content of up to 46.9%, which is comparable to *Chlorella vulgaris* (45.3%) and *Spirulina platensis* (50.4%), two alternative renowned superfoods (6). In addition, its crude polysaccharide content is 12.5%, its dietary fiber content is 11.9%, and it contains eight essential amino acids, various trace elements, unsaturated fatty acids, and biotin, which are essential for human health (7). Secondary metabolites, unsaturated fatty acids, terpenes, and other nutrients produced during the fermentation render the algal taste unique, have a hypoglycemic effect (8), improve visual acuity, and enhance immunity (9, 10).

Although *Chlorella vulgaris* is highly nutritious, its cell wall is difficult to digest, which leads to low nutrient absorption (11). On the other hand, *Spirulina platensis* is usually produced in runway ponds, which are susceptible to contamination by stray bacteria that can affect its quality (12). In contrast, the cell wall of *C. reinhardtii* has a multilayered polysaccharide-protein structure that enables easy digestion and absorption of the alga in the human body (13), and its production involves a cleaner process of heterotrophic fermentation. Due to its high nutritional value, *C. reinhardtii* shows considerable potential in fields such as alternative protein raw materials, pharmaceuticals, brewing industry, food additives, and animal feed. Through sustainable and efficient cultivation, this alga may alleviate the increased demand for more conventional land-crop resources (14).

In industrial production, an algal strain is often modified according to product requirements to improve its productivity (15). Mutagenesis is a common technique that usually involves methods such as ultraviolet mutagenesis, ionizing radiation, base analogs, or the addition of alkylating agents. Atmospheric and room temperature plasma (ARTP) is an emerging microbial mutagenesis technology that was first employed to microbial breeding in 2010 (16). Since 2020, the number of research reports and citations regarding ARTP mutagenesis technology has increased significantly, indicating that it is being discussed more frequently by researchers (17). The ejection temperature of ARTP is close to room temperature, and the discharge is more uniform, ensuring increased micro-organismal survival rates (18). The plasma jet contains chemically active particles, such as the excited states of He, O, N_2_, and OH, which can penetrate the cell wall and cell membrane and damage the DNA (19). It activates the DNA repair mechanism in cells. Repairing severe DNA damage is prone to phenomena such as base mismatch, deletion, and frameshift, leading to numerous gene mutations (20). Compared with traditional mutagenesis, the ARTP instrument is easy to use and enables fast sample preparation; it damages the DNA in a unique way, and there are many induced mutation sites. In addition, no chemical pollutants or toxins are emitted during use. Electromagnetic pollution is well below the international standards, making it safe for operators to use without requiring any special protection. Despite these advantages, it is difficult to apply this method in plants and animals.

ARTP mutagenesis has been successfully applied to microalgae. For example, the high-protein yellow mutant A4-1 (21) obtained from *Auxenochlorella pyrenoidosa* represents a high-quality algal strain for fermenting and producing alternative proteins. An algal strain (M8) with high lipid production was obtained from *Parachlorella kessleri*, and its fatty acid composition complies with the standards of high-quality biodiesel (22), which could help alleviate pressure on the reserves of traditional fossil energy. A mutant (A4) with 1.8–5.2× higher H_2_ production in *C. reinhardtii* enables the production of low-energy-consumption and environmentally friendly hydrogen through water photolysis mediated by photosynthesis (23).

Additionally, chlorophyll deficiency in the alga reduces its grassy flavor (24). We employed ARTP mutagenesis technology to treat the wild-type *C. reinhardtii* CC-1690 (*21gr*) variant, a strain that deviates from conventional green algae. We screened for chlorophyll-deficient mutants under dark light conditions. This alga can exist in the dark and steadily proliferate over an extended period of time using acetate as a carbon source, which proves its versatility, with additional potential applications in food, healthcare products, pharmaceuticals, and cosmetics.

## 2 Materials and methods

### 2.1 Microalgae materials

Wild-type *Chlamydomonas reinhardtii* CC-1690 (*21gr*) was purchased from the *Chlamydomonas* Resource Center (University of Minnesota) and subcultured in our laboratory.

### 2.2 Algae strain culture

A tris-acetate-phosphate (TAP) (25) medium was configured in a 250 mL cultivation bottle, with high-temperature autoclave sterilization. A small amount of the algae was inoculated into a sterile medium, aerated, and incubated in photoperiods (light: dark cycle 14 h: 10 h, 144 μmol∙m^−2^∙s^−1^) at 24°C for 72–96 h until the cells reached the logarithmic growth phase. The cells were then placed into sterile centrifugal tubes and spun at 1,200 g for 3 min on an ultra-clean workbench. Microalgal pellets were washed thrice with deionized water and then diluted to 5 × 10^6^ cells mL^−1^ with deionized water for ARTP mutagenesis.

The algae were spread on TAP agar plates and cultured in darkness or different photon flux densities with a spotlight on the top at 54 μmol∙m^−2^∙s^−1^, 144 μmol∙m^−2^∙s^−1^, and 360 μmol∙m^−2^∙s^−1^.

### 2.3 ARTP mutagenesis

When the lethality rate reaches 90%, the mutation rate of the algal strain is relatively high and the reversion mutation is reduced; however, the lethality and mutation rates are not positively correlated (26). The wild-type strain *21gr* was treated with a time gradient (0, 5, 10, 15, 20, 30, 40, 50, 60, and 90 s) and then added to the TAP medium in an orbital shaker (Zwy-240; Zhicheng Analytical Instrument Manufacturing Company, Shanghai, China) for 100 r/min at 24°C for 8 h. The cells were then collected by centrifugation at 1,200 g for 3 min (Avanti J-E centrifuge; Beckman Coulter Incorporation, United States); 1 mL of the TAP medium was added to resuspend them, and the solution was then placed onto agar plates at 24°C for 5–7 days in photoperiods. All experimental trials were performed in triplicate. Surviving algal monoclone were counted and lethality was determined (27), using the equation (1):


(1) $$F=\left(1-\frac{N_{1}}{N_{0}}\right)\times 100\%$$

In this equation, F represents the “lethality rate,” N_0_ is the “number of the control plate algae,” and N_1_ is the “number of the treated plate algae.”

A 10 μL sample of the diluted algal liquid was spread evenly over the surface of a sterile TAP plate medium; the plate was then transferred to the operating room of the ARTP breeders (ARTP-M mutualized breeding instrument; Tianmu Biotechnology Company, Wuxi, China) and placed in a corresponding groove using sterile tweezers. The equipment parameters were power (120 W) and gas volume (10 SLM), with the optimal treatment time determined by trial tests. After the carrier was drawn into the eppendorf (EP) tube with 1 mL TAP medium in an orbital shaker for 1 min. Then, the cells were transferred to a 50 mL centrifuge tube with a fresh TAP medium, wrapped in tin foil, and shaken (100 r/min) at 24°C for 8 h. The recovered algal cells were centrifuged at 1,200 g for 3 min and then evenly plated onto TAP agar plates.

### 2.4 Isolation of the chlorophyll-deficient mutants

The TAP agar plates were completely protected from light at 24°C. After at least 7 days, the appearance of algal colonies was carefully observed under dim light. The non-green colonies were removed and placed onto fresh TAP plates for complete dark culturing at 24°C. This screening operation was repeated more than 5 times until the phenotypic was stable, preserve them in TAP plates and liquid nitrogen, and preserve in TAP plates activated once a week to ensure the growth vitality of algae.

### 2.5 Cloning of the mutation site

The entire genomic DNA of both the *21gr* and *A7S80* mutant strains was recovered using cetyltrimethylammonium bromide (CTAB) (28), extracted with PCI (50% v/v phenol; 48% v/v chloroform; and 2% v/v isoamyl alcohol), mixed by inverting, and then centrifuged at 18,000 g for 10 min. The aqueous layer was transferred to a new tube with PCI (96% v/v chloroform and 4% v/v isoamyl alcohol), mixed by inverting, and centrifuged again at 18,000 g for 10 min. The aqueous layer was then transferred to a new tube with 0.7 volume of isopropanol to precipitate the DNA for 20 min at −20°C; the DNA was then pelleted by centrifugation at 18,000 g for 10 min. The pellet was washed thrice with 70% ethanol, after which 80 μL of a TE buffer was added to dissolve the pellet to obtain the genomic DNA.

Using the genomic DNA as the template, polymerase chain reaction (PCR) (Veriti 96-Well PCR instrument; Thermo Fisher Scientific Incorporation, United States) amplification was performed using Phanta Super-Fidelity DNA Polymerase (Nanjing Vazyme Biotech Co., Ltd). The amplification reaction program involved pre-denaturation at 95°C for 2 min; denaturation at 95°C for 10 s, annealing at 60°C for 30 s, and an extension at 72°C (30 s kb^−1^) for 35 cycles; and a final extension at 72°C for 10 min. After electrophoresis on a 1.5% agarose gel, the target fragments were excised and sent to Sangon Biotech (Wuhan) Co., Ltd. for sequencing. The sequences of the mutant strains were compared with those of the wild-type algal strain.

### 2.6 Algal cultivation

The seed for heterotrophic growth was cultured in a 500 mL Erlenmeyer flask placed in an orbital shaker at 24°C and 150 r/min for 72 h. This culture was used to inoculate a 5 L fermenter (Biotech-5JG fermenter, Baoxing Bio-Engineering Equipment Co., Shanghai, China). The primary fermentation broth had a TAP medium maintained at 26 ± 0.3°C, with a pH value of 7.8 ± 0.2 (pH sensor, Mettler Toledo, Switzerland). This pH balance was achieved and maintained by adding 20% v/v acetic acid or 0.5 mol L^−1^ sodium hydroxide. The feeding medium consisted of 50 × TAP, concentrate without tris base, and was supplemented with 0.5 mol L^−1^ sodium acetate and 20% v/v acetic acid. Additionally, corn oil with 30% m/m triglyceride was used as a defoamer (29).

For the seed, the wild type and the mutant grown on the TAP plates were inoculated in a liquid TAP medium in an orbital shaker at 24°C and 150 r/min; the mutant was cultured in darkness. Flame inoculation into the fermenter was performed when the optical density (OD) reached 0.6–1.0; this initial algae density was approximately 10^5^ cells mL^−1^. The fermenter parameters were set as follows: temperature at 26 ± 0.3°C, pH at 7.8 ± 0.2, initial agitation rate at 100 r/min, air inlet flow rate at 4 L min^−1^, and dissolved oxygen above to 30%. Feeding commenced after 48 h in accordance with equation (2). Foam was monitored after 12 h of feeding, after which the defoamer was added; the fermentation time depended on the tank biomass, but it was approximately 144–168 h. The fermentation broth was centrifuged at 4,200 g for 5 min. The algal mud was refrigerated at −80°C for pre-cooling. The sample was dried using a freeze dryer lyophilizer (ZLGJ-18, Ningbo Scientz Biotechnology Company, China) until no obvious surface moisture remained. The *C. reinhardtii* powder was obtained by grinding and sieving.


(2) $$v=v_{0}\times e^{u\times \left(t-t_{0}\right)}$$

In this equation, “v” represents the fermentation process feeding rate (mL h^−1^), “v_0_” represents the initial feeding rate (mL h^−1^), “e” is a natural constant, “u” is the specific microbial growth rate, “t” is the fermentation time, and “t_0_” is the initial feeding time.

### 2.7 Protein content

The protein content was determined using a modified Lowry method (30, 31). The sample was diluted with distilled water in an ultrasonic cell pulverizer (JY 92-IIDN, Ningbo Scientz Biotechnology Company, China). A sample of 1 mL was added into an EP tube with 0.9 mL of solution A (100 g L^−1^ of sodium carbonate, 2 g L^−1^ of potassium sodium tartrate, and 0.5 mol L^−1^ of sodium hydroxide). This mixture was then incubated at 50°C for 10 min and cooled to room temperature. Then, 1 mL of solution B (0.2 g L^−1^ potassium sodium tartrate, 0.1 g L^−1^ copper sulfate pentahydrate, and 0.1 mol L^−1^ sodium hydroxide) was added and mixed well and was left for 10 min. A 3 mL sample of solution C (1/16 v/v Folin–Ciocalteu phenol aqueous solution) was added, and then, the sample was incubated at 50°C for 10 min. The standard curve was plotted with bovine serum proteins (0, 0.200, 0.400, 0.600, 0.800, and 1.000 g L^−1^), with absorbance measured at OD_650_. The sample protein contents were estimated from the standard curve.

### 2.8 Crude polysaccharide content

The crude polysaccharide content was determined using a phenol-sulfate acid method (32–34). A homogeneous lyophilized algal powder (0.500 ± 0.001 g) was added to 15 mL of deionized water; then, the solution was extracted by microwave at 140°C for 2 h, boiled, and kept without liquid flow. Then, after adding 5 mL of water, the solution was stirred and cooled to room temperature; 75 mL of anhydrous ethanol was added to the solution, and the solution was maintained at 4°C overnight, before being centrifuged at 4,800 g for 10 min. The supernatant was discarded, the residual liquid was evaporated with heat, the sample was diluted to 250 mL, and the middle filtrate was collected. The standard curve was prepared using a 100 mg L^−1^ glucose standard solution (0, 0.200, 0.400, 0.600, 0.800, and 1.000 mL). The solution was diluted to 1 mL with water. After that, 1 mL of a 5% v/v phenol solution was added and mixed quickly, then 5 mL of sulfuric acid was added and mixed for 10 min, and finally, the solution was mixed and incubated with water at 30°C for 20 min. The absorbance was monitored at 490 nm; the concentration of the solution was determined using equation (3).


(3) $$X=\frac{m_{1}\times V_{1}}{m_{2}\times V_{2}}\times 0.9\times 10^{-6}\times 100$$

In this equation, “X” represents the crude polysaccharide content of the sample (g∙0.1 kg^−1^), “m_1_” is the sugar content in the assay solution corresponding to the standard curve (μg), “m_2_” is the sample mass (g), “V_1_” is the sample volume (mL), “V_2_” is the volume of the assay solution for colorimetric determination (mL), “0.9” is the conversion factor for glucose to dextran, and “10^−6^” is a conversion factor.

### 2.9 Total chlorophyll content

The chlorophyll concentration was determined by spectrophotometry (35, 36). Homogeneous lyophilized algal powder weighing homogeneous lyophilized algal powder 0.500 ± 0.005 g, added mixture, equal volume of 95% ethanol and 99.5% acetone mixed, for extraction. The volume was diluted to 100 mL, vortexed well, and kept still for 8 h. It was then filtered, and the absorbance was determined at OD_644_ and OD_662_. The chlorophyll content was determined using equation (4).


(4) $$F=\left(5.134\times A_{1}+20.436\times A_{2}\right)\times \frac{\nu }{1000\times m}$$

In this equation, “F” represents the total chlorophyll content (mg g^−1^), “A_1_” is the absorbance value of the test solution at 662 nm, “A_2_” is the absorbance value of the test solution at 644 nm, “m” is the specimen mass (g), and “v” is the test solution volume (mL).

## 3 Results

### 3.1 ARTP mutagenesis of the *Chlamydomonas reinhardtii*

**Figure 1:**
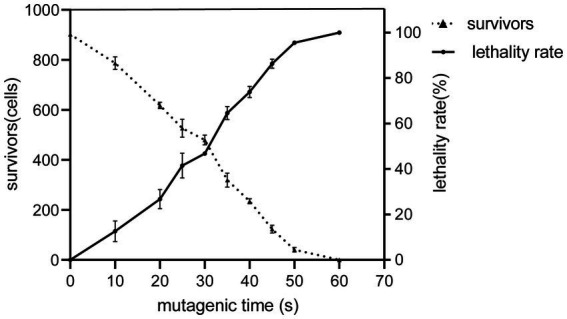
ARTP mutagenesis and lethality of the wild type *21gr* at different irradiation times. The solid line shows that the mortality rate increased gradually with the extension of the reaction time. The dashed line shows a gradual decline in survival over time.

The number of algae that survived the ARTP treatment at different times was counted to determine the lethality rate. The relationship between the mutagenesis time and lethality rate is shown in Figure 1. For a mutagenesis time of 45 s, the lethality rate was 86.29%, and for ≥60 s, it was approximately 100%. Accordingly, we used 45 s as the optimal time for the ARTP mutagenesis of *C. reinhardtii*.

### 3.2 Pigment profile of the chlorophyll-deficient mutants in different light intensities

**Figure 2:**
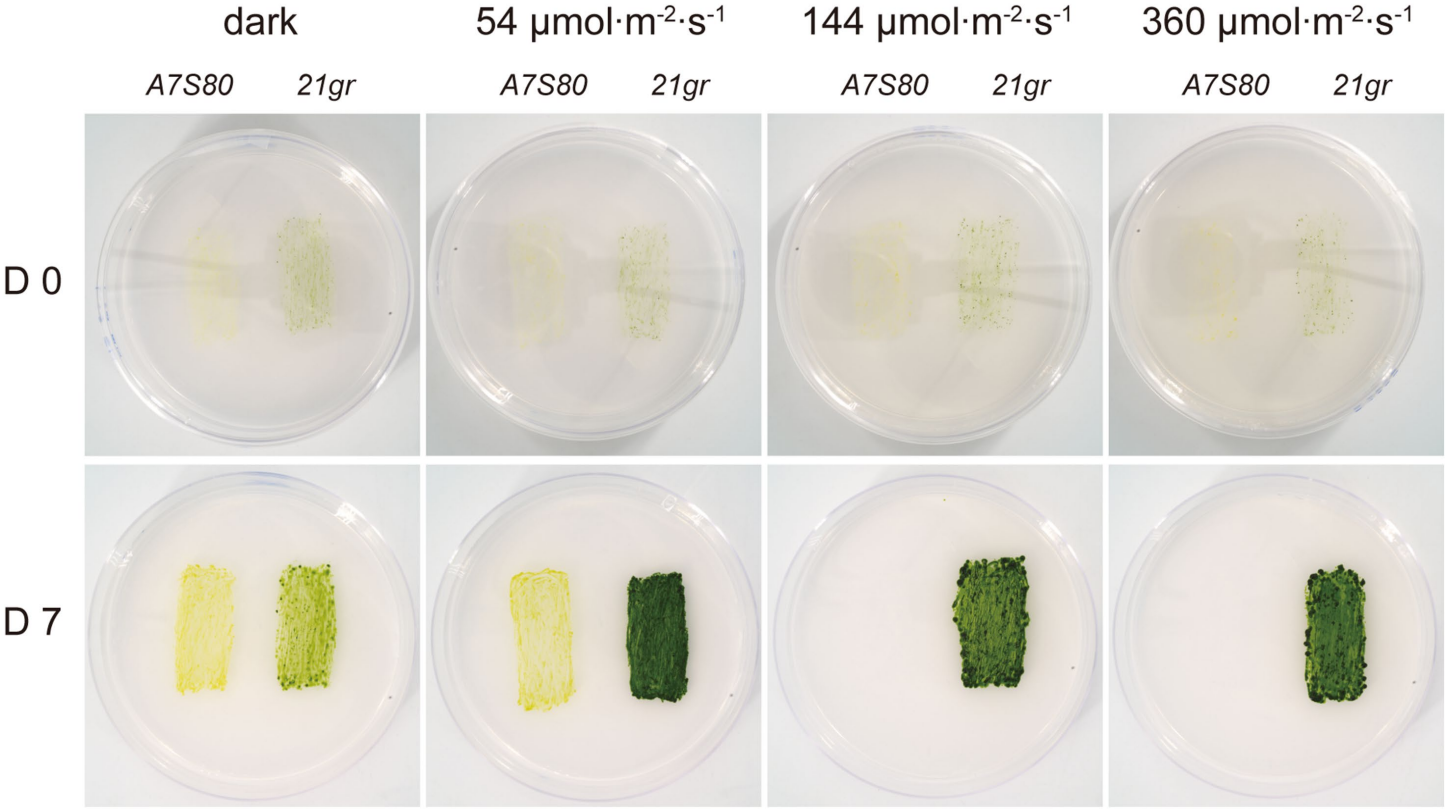
Growth and pigmentation of the mutant. The wild-type sequences were shown for control. After inoculation, the plates were incubated for 7 days in the dark and at different light intensity environments. The yellow mutant survived on acetate as a carbon source under dark light but turned white and died as the light intensity increased.

After multiple rounds of the ARTP mutagenesis and dark selection, a yellow colony, which we named *A7S80*, was isolated (Supplementary Figure S1). The yellow coloration indicated a chlorophyll deficiency. We selected and isolated this variant in the absence of light (using acetate as a carbon source) to suppress the demand for energy via photosynthesis. This mutant remained yellow at low light (54 μmol∙m^−2^∙s^−1^), and its growth was inhibited at light intensities of 144 μmol∙m^−2^∙s^−1^ and 360 μmol∙m^−2^∙s^−1^ (Figure 2).

To better understand the differences in growth between the *A7S80* and *21gr* strains when exposed to different light conditions, a series of liquid cultures of both strains was examined (Supplementary Figures S2, S3). At 54 μmol∙m^−2^∙s^−1^, the growth rate and final concentration of *21gr* were significantly higher than those of *A7S80*. At 144 μmol∙m^−2^∙s^−1^, *A7S80* survived, and its growth rate was slightly slower than that under the light condition of 54 μmol∙m^−2^∙s^−1^, but on day 5, its growth rate suddenly increased, accompanied by a gradual change in color from yellow to green—a phenomenon also reported for Chinese cabbage and *C. reinhardtii* (37, 38). At 360 μmol∙m^−2^∙s^−1^, *A7S80* could not survive and its cells broke down. Under dark conditions, the growth rate of *A7S80* was slightly higher than that of *21gr*. These phenomena indicated that *21gr* was better adapted to light and *A7S80* to darkness.

### 3.3 Mutation characterization

There are few reports of edible “non-green” *C. reinhardtii*. Color variation is mainly caused by a defect in chlorophyll synthesis. In all photosynthetic organisms, the biosynthesis step of protoporphyrin IX (PIX) only occurs within the chloroplast, with PIX being the precursor of chlorophyll and heme (39, 40). Magnesium ion chelatase has three subunits, CHLD, CHLI, and CHLH. Two AAA+ subunits form the CHLD complex (41, 42), which interacts transiently with the body region of the CHLH protein through the C-terminal integrin domain of CHLD and then hydrolyzes ATP, driving the conformational change of the CHLH-porphyrin complex and promoting the insertion of Mg^2+^ ions into the PIX ring to form magnesium protoporphyrin IX (MgPIX) (43, 44). MgPIX methyltransferase (*chlM*) is another key rate-limiting enzyme in the chlorophyll-synthesis pathway, catalyzing the transfer of the methyl group from S-adenosylmethionine to the carboxyl C13 propionate side chain of MgPIX (45). After accepting the methyl group, MgPIX undergoes cyclization and forms protochlorophyll under the action of divinyl reductase (46). Furthermore, chlorophyll a or chlorophyll b is formed under the action of protochlorophyll oxidoreductase (*chlL*, *chlN*, and *chlB*) (47).

Two mutants with a yellow and orange color for which mutant alleles were identified in the *chlH* coding region have been reported (48); two more yellow mutant alleles have been identified in the *chlM* gene (49), and a dark yellow mutant for which the mutation site was localized in the *chlL* gene has also been reported (50). Combining the chlorophyll synthesis pathway and these findings of non-green mutants, we considered that *chlD*, *chlH*, *chlI*, and *chlM* were the main candidate mutant genes. The primers for the PCR amplification and the primer sequences for the related genes are shown in Table 1.

**Table 1:** Analysis of the non-green trait gene information.

Gene	Gene ID	Functional description	Primer sequence
*chlD*	Cre05.g242000	Mg chelatase D subunit	*chlD-*S:CTCGGAAGTCGGAGCAATAAT *chlD-*AS:CGGAGGTAGGTGGAAAGCAAT
*chlH*	Cre07.g325500	Mg chelatase H subunit	*chlH-*S:TCAGCGGATCTTTACTT *chlH-*AS:TCGGGCACAAACACT
*chlI*	Cre06.g306300	Mg chelatase I subunit	*chlI-*S:CGCTAGGGAACTGAAATCG *chlI-*AS:TCGGAGACGGGTGACATT
*chlM*	Cre12.g498550	Mg-protoporphyrin IX methyltransferase	*chlM-*S:TCAGGTGCCTCCGTTAC *chlM-*AS:CGGCTTGATTGTTTCG

S represents the sense primer, and AS represents the anti-sense primer.

**Figure 3:**
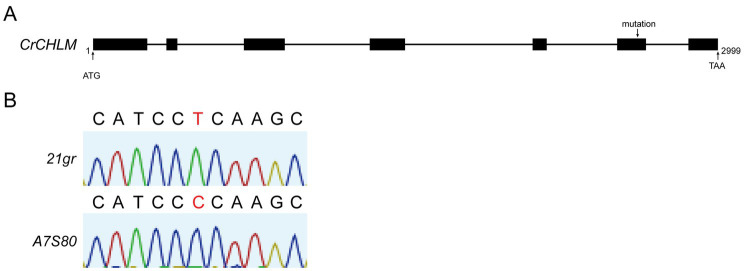
**(A)** Structure of the *chlM* gene, the PCR fragments were sequenced respectively, and aligned with wild type sequences. **(B)** The black letters represent the ribonucleotide signals with no differences, and the red letters represent the ones with differences.

The mutations in the gene were identified by sequencing the fragments generated with the gDNA of *21gr* and *A7S80* (Figure 3A). Regarding *A7S80*, a “T” to “C” single base mutation occurred in exon 6 of the *chlM* gene at the 95^th^ base position as compared with *21gr*, resulting in the mutation of leucine to proline (Figure 3B).

### 3.4 Fermentation yield and nutritional components

**Figure 4:**
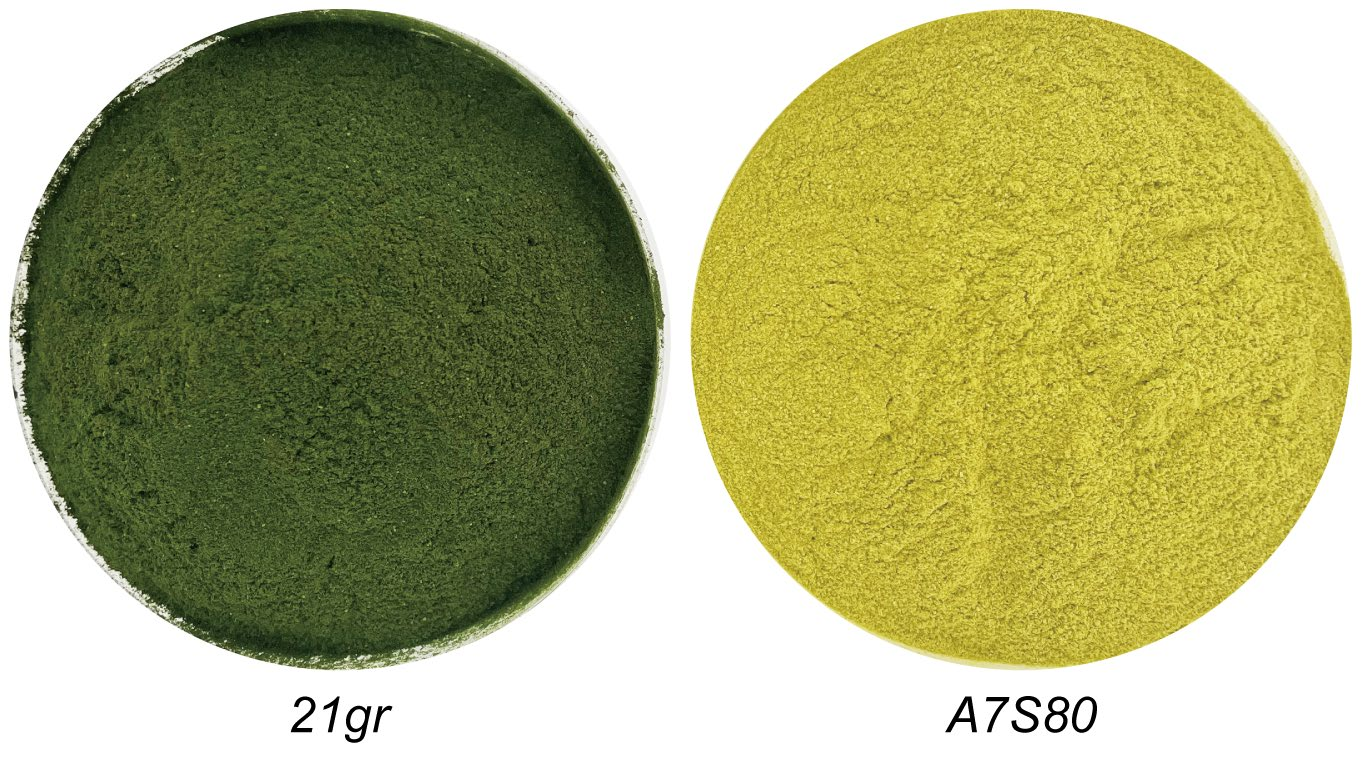
Different pigments of *21gr* and *A7S80*, grown in the fermentation for 7 days and freeze-dried with green and yellow colors.

After 144 h of fermentation, 3.5 L of the “algal liquid” was collected. Following centrifugation and freeze-drying, dried algal powders of *21gr* and *A7S80* were prepared (72 g and 76 g, with yields of 20.57 g L^−1^ and 21.7 g L^−1^, respectively). The previous highest concentration of wild-type *C. reinhardtii* achieved in a fermented culture was 25.44 g L^−1^ (51), but this required 237 h; we achieved a higher output per unit time. We cultured algae in 200 L and 2 T fermenters and achieved a maximum yield of 18.26 g L^−1^, which was slightly lower than that in a 5 L fermenter. This difference might have been caused by an ineffective defoamer, which limited the rotation speed and led to low dissolved oxygen levels, causing the plateau phase to arrive earlier. An alternative defoamer with a low inhibitory effect and excellent defoaming performance is required. Compared with the *21gr* powder, the *A7S80* algal powder was bright yellow in color (Figure 4). The protein, crude polysaccharide, and chlorophyll components of *21gr* and *A7S80* were simultaneously detected. Although the protein and crude polysaccharide contents in *A7S80* were slightly lower, the differences were not significant; the chlorophyll content in *A7S80* was significantly lower. The slight difference in the protein content might have been caused by the mutation (Table 2).

**Table 2:** Composition of the nutrients of the *21gr* and *A7S80* freeze-dried powder.

Algae strain	Proteins (g/100 g)	Rough polysaccharide (g/100 g)	Chlorophyll (mg/g)
*21gr*	50.55 ± 1.56^a^	12.04 ± 0.59^a^	20.53 ± 0.56^a^
*A7S80*	47.79 ± 1.42^a^	10.48 ± 0.8^a^	0.16 ± 0.01^b^

Different letters indicate significant differences (*p* > 0.05) between the strains and treatments. Values are given as means ± standard deviation for the three biological replicates (*n* = 3). Different letters of the superscript indicate significant differences (*p* > 0.05) between the strains.

## 4 Discussion

In the early stages of microalgal industry development, attention was focused on healthcare products derived from *Chlorella vulgaris* and *S. platensis*. During this period, technological and economic environments were very different. Culture conditions and other factors, such as smell, reduced the market demand for the product. The cost of *Chlorella* powder has increased due to post-processing steps, such as wall breaking and defishability (52). The current price of the algae is still not affordable for the general public, making it difficult for most people to benefit from it.

*Chlamydomonas reinhardtii* has been used as a model organism in biological research for decades. Its genome has been sequenced, its genetic background is clear, its culture and related technical systems are established, its cell wall is more readily broken down in the human gut, and it smells significantly less fishy than *Chlorella*. These attributes are conducive to both its processing and its use as a food resource. In addition, because of its reduced odor, high protein content, rough polysaccharides, and other nutrient contents, *C. reinhardtii* was approved as a new food ingredient by the National Health Commission of China in 2022. Although the development of products remains in its infancy, products incorporating this alga, such as pastries, yogurt, and milk tea, have already been marketed.

Microalgae contain a variety of antioxidants such as carotenoids, phenols, flavonoids, polyunsaturated fatty acids, vitamins, phycobilin coenzyme Q, and other compounds composed of peptides. These chemicals have anti-cancer properties (53). High doses of ascorbic acid significantly impede cancer growth, whether administered alone or with conventional anticancer drugs, but they should be administered intravenously; ascorbic acid taken orally only leads to modest increases in plasma concentration (54). Cancer cells are destroyed by epigenetic regulation, such as DNA and histone demethylation, and reconstitution of 5-hydroxymethylcytosine by oral vitamin C supplementation (55). Biomaterials composed of living microalgae and biocompatible components have unique physiological characteristics, such as photosynthetic activity, autofluorescence, and autonomous movement. By encapsulating microalgae in conventional biomaterials to maintain their photosynthetic activity, these materials can provide local oxygen and serve as biocompatible interfaces to regulate cellular activities. In addition, the autonomous movement of microalgae has influenced the development of biohybrid microrobots. These microrobots through precisely controlled movements can deliver drug molecules to target areas for therapeutic purposes (56).

Cosmeceuticals are cosmetics that have both cosmetic and pharmaceutical applications. Cosmeceuticals are often used in dermatology to enhance skin tone and skin shine, as well as to provide anti-aging effects. The cosmeceutical industry is a growing and highly profitable industry globally (57). Microalgae contain various active substances, such as phosphophenols, polysaccharides, and carotene, which can inhibit the activity of tyrosinase, reduce ultraviolet radiation damage, prevent melanin production, whiten skin, and reduce wrinkling (58). In addition, polysaccharides have moisturizing and mollifying effects (59), and the steroid fucosterol and PYP1-5 peptide can inhibit matrix metalloproteinase expression and promote collagen synthesis and elastin production (60, 61).

Microalgal biomass can be converted into biodiesel, bioethanol, biohydrogen, and bioactive substances and products, such as animal feed, biofertilizers, bioplastics, cosmetics, and pharmaceuticals. With the support of ARTP technology, high-yielding algal strains with targeted traits can be engineered to both improve production efficiency and save production costs. The cultivation of microalgae can also capture CO_2_, thereby reducing GHG emissions.

Although ARTP mutagenesis is a non-transgenic technology, it has shown great potential in energy and other fields, but the safety of mutant strains in food must be strictly evaluated. Combined with modern biotechnology, such as genomics, metabolomics, and epigenetics, how ARTP mutagenesis affects microalgal cell metabolic pathways can be better understood. Conditions to optimize mutagenesis can be developed and microalgal mutants that produce high yields of target products can be engineered. Through interdisciplinary research, the synergistic effect of ARTP mutagenesis and other breeding techniques can be explored to further improve the efficiency and effectiveness of microalgae breeding.

We used ARTP technology to directionally screen under dark conditions (Supplementary Figure S4), in which the yellow mutant *A7S80* grew using acetate as a carbon source, and the chlorophyll content and “green grass” taste were reduced. The nutrient contents of the protein and rough polysaccharide in *A7S80* were similar in values to those in *21gr*. Cultivated using a high-density fermentation technology developed by our laboratory, the biomass of *C. reinhardtii* can be significantly increased. This advancement enables us to quickly obtain large amounts of algal powder so that new food formulas can be developed to cater existing *C. reinhardtii* markets for human and animal food, healthcare products, drugs, fuels, and cosmetics.

## Data Availability

The datasets presented in this study can be found in online repositories. The names of the repository/repositories and accession number(s) can be found at: https://phytozome-next.jgi.doe.gov/info/CreinhardtiiCC_4532_v6_1.
