# Supplementary figures and images for: Mutagenesis selection and large-scale cultivation of non-green Chlamydomonas reinhardtii for food applications
Source: Front Nutr. 2024 Sep 25;11:1456230. doi: 10.3389/fnut.2024.1456230 (PMC11462061; doi:10.3389/fnut.2024.1456230)

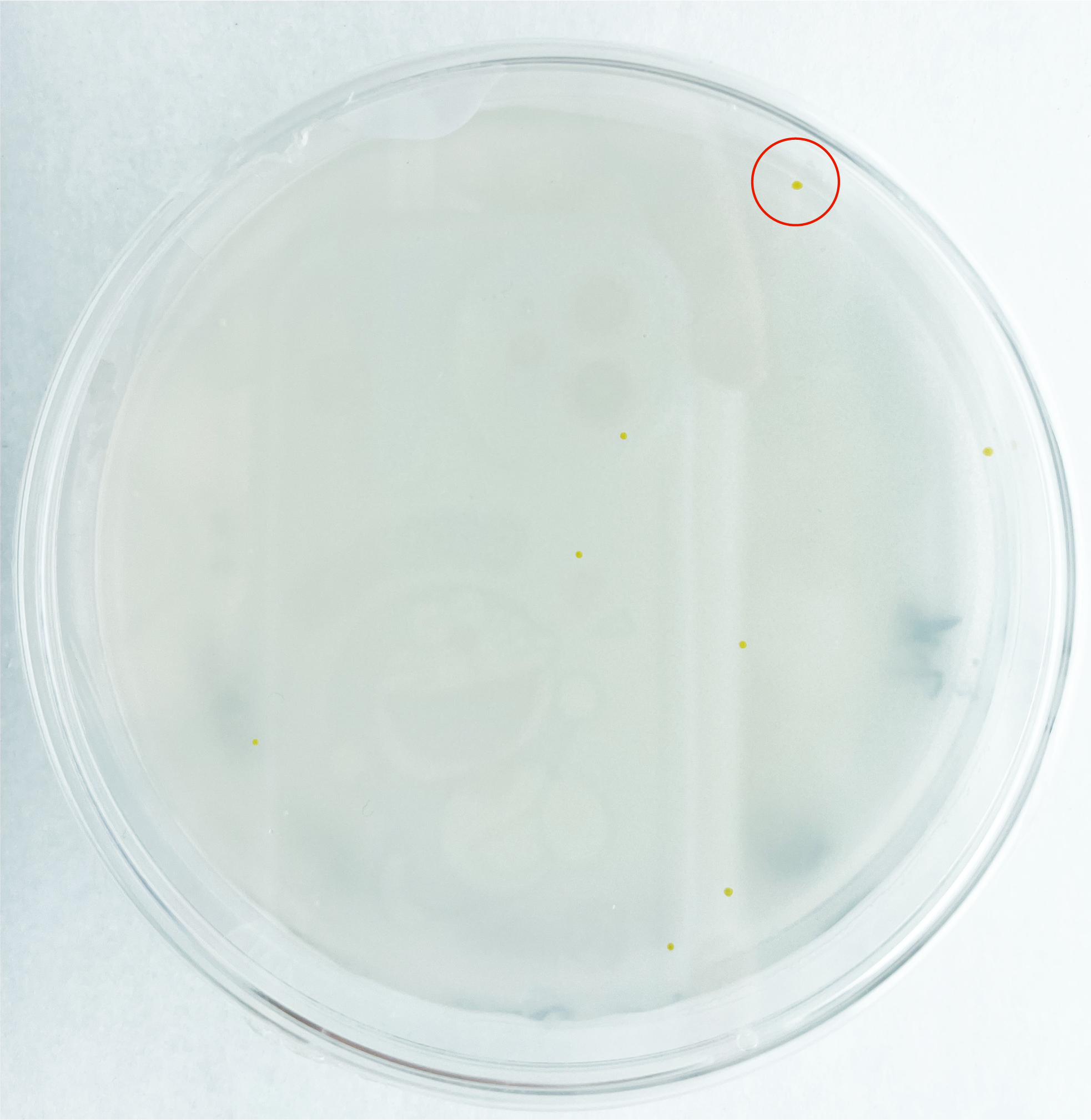

Supplement: Supplementary file 1 [file Image_1.TIF]

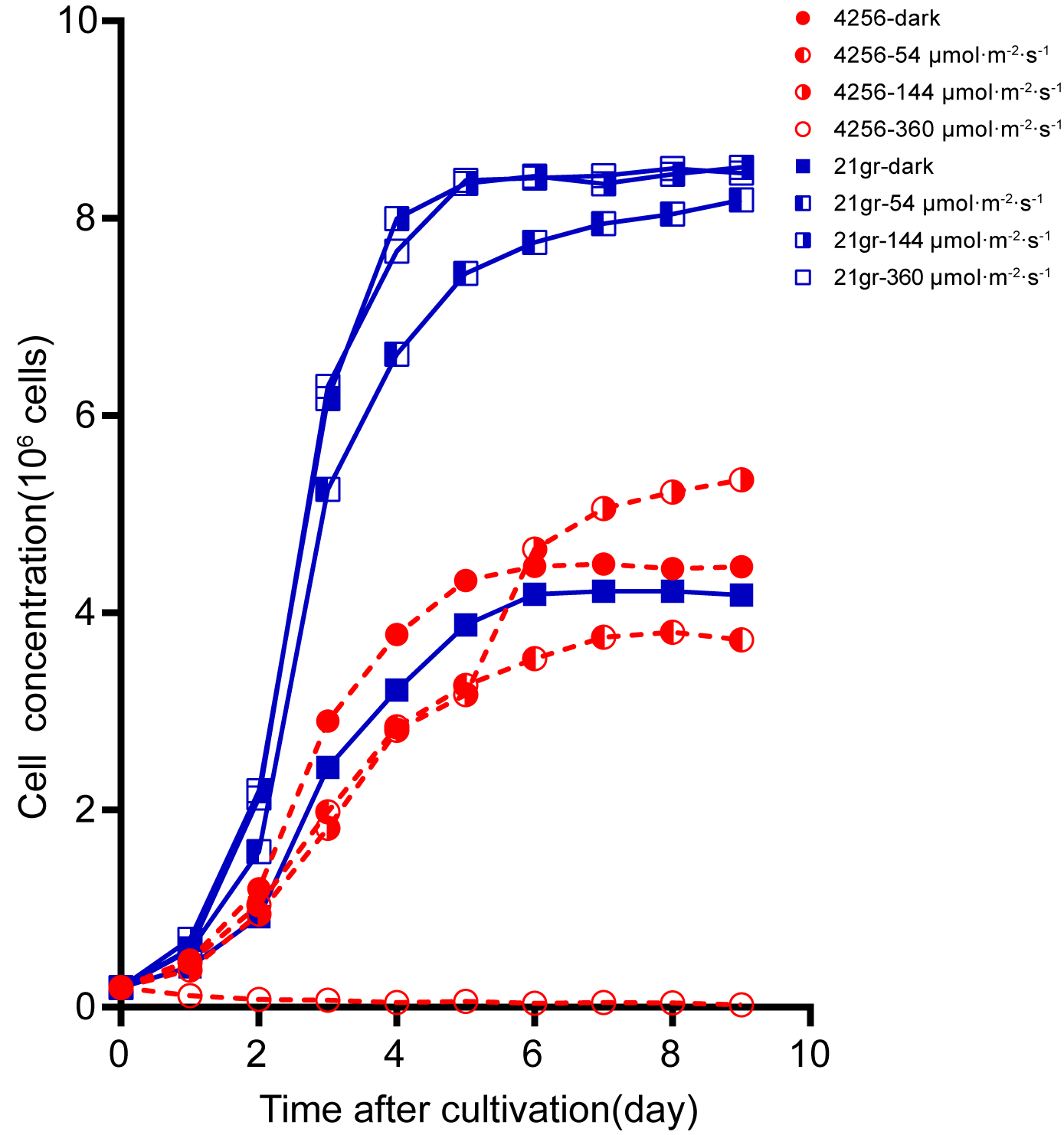

Supplement: Supplementary file 2 [file Image_2.TIF]

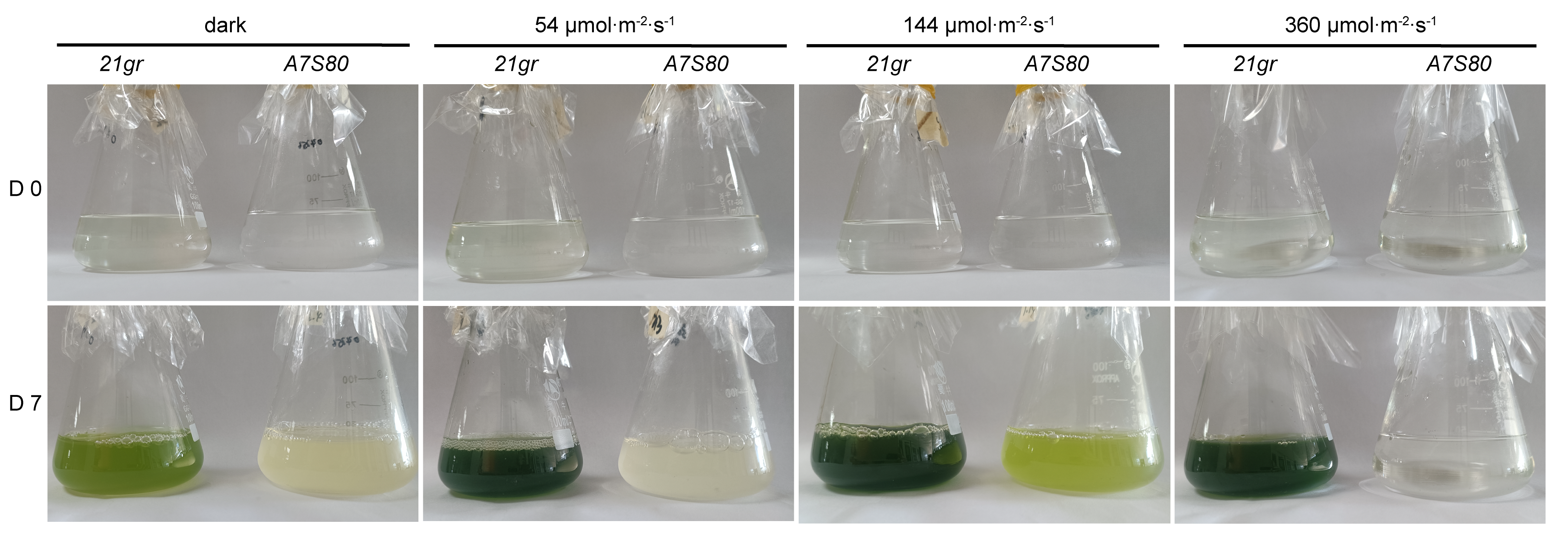

Supplement: Supplementary file 3 [file Image_3.TIF]

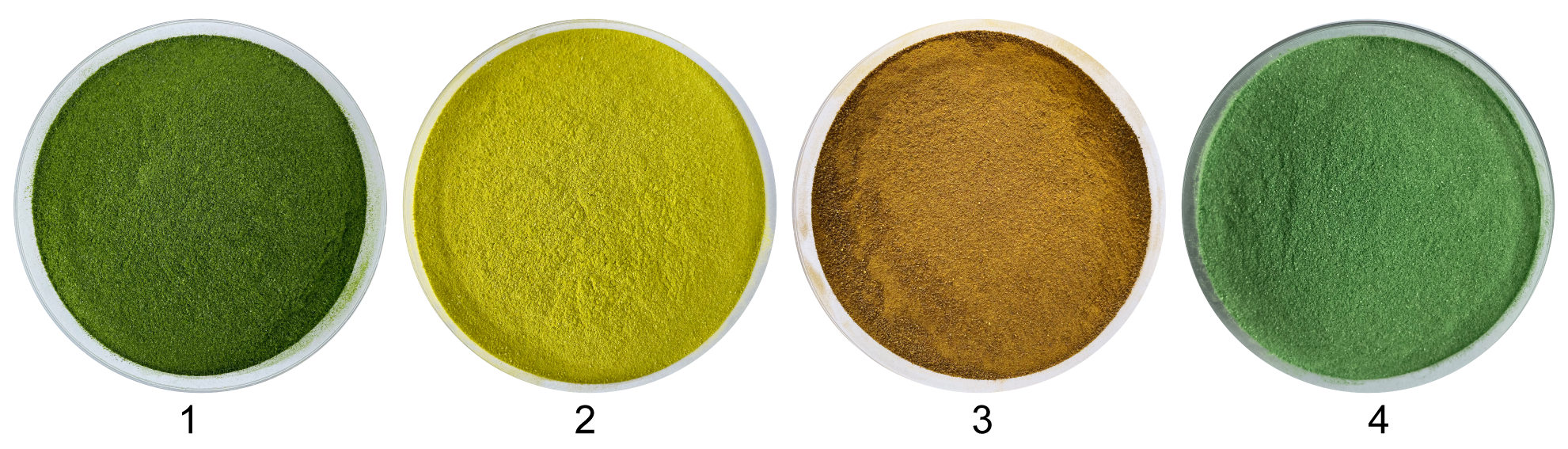

Supplement: Supplementary file 4 [file Image_4.TIF]
